# Influence of Physical Dimension and Morphological-Dependent Antibacterial Characteristics of ZnO Nanoparticles Coated on Orthodontic NiTi Wires

**DOI:** 10.1155/2021/6397698

**Published:** 2021-10-14

**Authors:** Mona Gholami, Mahdiyeh Esmaeilzadeh, Zahra Kachoei, Mojgan Kachoei, Baharak Divband

**Affiliations:** ^1^Department of Orthodontics, Faculty of Dentistry, Tabriz University of Medical Sciences, Tabriz, Iran; ^2^Student Research Committee, Faculty of Dentistry, Tabriz University of Medical Sciences, Tabriz, Iran; ^3^Polymer Division, Chemistry Department, School of Science, University of Tehran, Tehran, Iran; ^4^Dental and Periodontal Research Center, Faculty of Dentistry, Tabriz University of Medical Sciences, Tabriz, Iran; ^5^Department of Inorganic Chemistry, Faculty of Chemistry, University of Tabriz, Tabriz, Iran; ^6^Dental and Periodontal Research Center, Tabriz University of Medical Sciences, Tabriz, Iran

## Abstract

White spot lesions (WSLs) are one of the adverse effects of fixed orthodontic treatments. They are the primary sign of caries, which means inhibiting this process by antibacterial agents will reverse the procedure. The current study tested the surface modification of nickel-titanium (NiTi) wires with ZnO nanoparticles (NPs), as antimicrobial agents. As the morphology of NPs is one of the most critical factors for their properties, the antibacterial properties of different morphologies of ZnO nanostructures coated on the NiTi wire were investigated. For the preparation of ZnO nanostructures, five coating methods, including chemical vapor deposition (CVD), chemical precipitation method, polymer composite coating, sol-gel synthesis, and electrospinning process, were used. The antibacterial activity of NPs was assessed against *Streptococcus mutans* by the colony counting method. The obtained results showed that all the samples had antibacterial effects. The antibacterial properties of ZnO NPs were significantly improved when the specific surface area of particles increased, by the ZnO nanocrystals prepared via the CVD coating method.

## 1. Introduction

Orthodontic treatment, bonding brackets, and engaging arch wires are safe and innocuous ways to correct tooth irregularities. However, in a patient with poor oral hygiene, it may cause some problems. Brackets bonded on the teeth may be a retentive place for accumulation of bacterial plaque and result in caries and increase the risk of white spot lesion (WSL), which is one of the inescapable complications of fixed orthodontic treatments [1, 2]. Clinically, WSLs are the first sign of enamel demineralization. White and opaque appearance is due to the loss of crystal structure caused by an excessive scattering of light [3].

To prevent caries and decrease the risk of WSLs, we should first disrupt the demineralization cycle. One way to prevent demineralization is reducing acidogenic bacterial plaque aggregation [4]. *Streptococcus mutans*, the major microorganism of the oral cavity, plays a crucial role in the demineralization process. Thus, suppressing this microorganism can lead to a lower risk of dental caries [4–6].

In the 21^st^ century, massive evolutions in science and technology gave rise to a new field named nanotechnology [7]. Nanotechnology, which is about particles at the nanoscale, takes an essential part of these advances in various fields, including orthodontics. Regarding this, the development of novel metal oxide nanomaterials brings certain privileges [8].

Among numerous metal oxides, zinc oxide (ZnO) has extensive practical applications in the rubber industry, cosmetics, pharmaceutical production, ultraviolet (UV) laser, and electronic industry [9]. This widespread usage is due to various properties of wide-bandgap semiconductors (3.37 eV). Furthermore, having reasonable production cost manifolds the advantages and makes ZnO nanoparticles (NPs) a suitable choice in different fields [10, 11].

Several synthesizing methods are used to produce ZnO NPs, such as precipitation and coprecipitation method, thermal hydrolysis techniques, solvothermal and hydrothermal processing, sol-gel method, microemulsion method, physical vapor deposition, vapor condensation method, biosynthesis method, high-energy ball milling, spray pyrolysis, vapor solid liquid, and chemical vapor deposition [12–16].

The antibacterial nature of ZnO NPs has been described in previous reports [6, 17–24]. This excellent property depends on morphology, crystallinity, and size of particles [25]. ZnO NPs eliminate microorganisms through various mechanisms and show antimicrobial properties as follows: (a) reactive oxygen species (ROS) production [26, 27], (b) the destruction of cellular wall [28], and (c) the release of Zn^2+^ ions [29, 30]. ROS production by metal oxide NPs is the most reported mechanism responsible for the antimicrobial effect, which can ruin the cellular integrity including DNA, proteins, and phospholipids [27]. The size and morphology of NPs have been expressed in several studies as influential factors in their antimicrobial properties [25, 31]. The morphology control of the ZnO NPs is one of the most critical items for their synthesis; thus, the reaction parameters should be regulated to ensure the size and shape of the product [32]. Besides, reducing friction is the reason for altering orthodontic NiTi wires' surface with these precious NPs [17, 33]. These two factors make ZnO-coated NiTi wires a charming alternative in modern orthodontics.

Different types of morphologies have been reported for ZnO NPs. The spherical, nanofiber, rod-shaped hexagonal, star-shaped, rod-shaped, and porous ball ZnO particles are among these NPs [34–36]. Several reports have shown different antimicrobial effects for these various morphologies [34, 37].

Overall, surface modification of NiTi wires with ZnO NPs was performed in previous studies to evaluate both antibacterial and tribologic characteristics of these NPs [6, 17]. However, as far as the researchers investigated, no study has evaluated the morphological differences of ZnO NPs synthesized by different coating methods and their morphology-dependent properties. Hence, this study was aimed at coating ZnO NPs on orthodontic wires using five different methods and comparing their antibacterial activity due to their various microscopic morphologies.

## 2. Materials and Methods

### 2.1. Materials

Potassium hydroxide and ethanolamine (MEA) were obtained from Merck, Germany, and polyvinyl alcohol (PVA) (average Mw = 13000-23000), zinc nitrate hexahydrate ≥ 98%, maleic anhydride 99%, zinc acetate dihydrate ≥ 98%, isopropanol ≥ 99.5%, and polyvinyl pyrrolidone (PVP) (average MW = 400000) were purchased from Sigma-Aldrich, Germany.

### 2.2. Synthesis Methods

Commercially, round 0.016-inch orthodontic nickel-titanium (NiTi) wires (OrthoTechnology, FL, USA) were cut to 18 cm pieces and cleaned by ultrasound with ethanol solution for 10 min at 30°C and then immersed in a 4 M potassium hydroxide at 100°C for 30 min under reflux. This makes the surface of the NiTi substrates more corrugated and results in better film adhesion.

The following techniques were used to develop different morphological ZnO NPs.

#### 2.2.1. Chemical Precipitation Method

The wires were immersed in a solution of 0.1 g zinc nitrate (Zn (NO_3_)_2_) in distilled water (50 mL), following dropwise addition of aqueous ammonia to make it alkaline under vigorous mixing condition. This makes the pH of the resultant solution 12. When the solution's temperature reached 50°C, the ZnO NPs were synthesized on the wires [17].

#### 2.2.2. Chemical Vapor Deposition Method

Chemical vapor deposition (CVD) process was done by using a CVD 420 Full, double-zone electric tube furnace (Nano sat Co, Semnan, Iran). The furnace has a quartz tube with 50 cm length and 5 cm internal diameter. Using a simple CVD method, ZnO thin films were grown on NiTi wire substrates. Oxygen gas and high-purity metallic Zn powders (99.999%) were used as oxygen and Zn sources, respectively. Next, at the center of the furnace, 0.3 g of Zn powder in a quartz boat was placed. At a distance of 1 cm from the downstream side of the source, the wires were placed horizontally. The whole system was pumped to a base pressure of about 10 Pa by a mechanical pump. High-purity Ar carrier gas was passed at a flow rate of 100 sccm, and oxygen gas was introduced into the reaction chamber at a flow rate of 30 sccm. The growth temperature in the reaction chamber was kept at 650°C for 30 min.

#### 2.2.3. Polymer Composite Coating

In this study, 100 mL aqueous polyvinyl alcohol (PVA) solution (5% *w*/*v*) was prepared by heating the solution at 70°C under constant stirring. Next, 1.5 g of maleic anhydride was mixed with the PVA solution using a mechanical stirrer (1000 rpm) at 70°C for 2 hours. Then, 0.1 g of nano-ZnO obtained by precipitation synthesis was added to the solution while stirring. The wires were immersed into it, and polymer coating containing ZnO NPs was coated on the wires. The film was dried at ambient temperature and subsequently cured in the oven at 100°C for 90 min.

#### 2.2.4. Sol-Gel Method

ZnO NPs were grown by a sol-gel synthesis technique on NiTi wires. The sol was prepared by dissolving 0.22 g of zinc acetate dihydrate in 9.49 mL isopropanol. This was followed by adding 0.06 mL ethanolamine gradually. As the Zn precursor, we used zinc acetate dihydrate, and isopropanol was used as the solvent with ethanolamine as the stabilizer. Then, to clarify the turbid solution, it was magnetically stirred for 2 h at 60°C, and the obtained solution was stored for 24 hours at room temperature. NiTi wire was dip-coated in the solution for 1 min and subsequently placed in the oven for 10 min to promote the solvent's rapid evaporation. The dip-coating process was repeated. This step was followed by drying the coated wafer at 100°C for 10 min and subsequent baking at 400°C for 1 h.

#### 2.2.5. Electrospinning Process

The electrospinning process was performed using Electroris (FNM Ltd., Iran, http://fnm.ir/) as an electrospinner apparatus having a high voltage and a syringe pump controllable in range of 1–35 kV and 0.1–100 mL/h, respectively. First, 0.38 g of zinc nitrate was mixed with 1.5 mL of distilled water, placed in an ultrasonic bath for 15 min, and then magnetically stirred for 5 h. The polymeric solution of PVP was prepared via mixing 1 g of PVP with 1.5 mL of ethanol and then magnetic stirring for 5 h. Then, zinc nitrate solution was added to the PVP solution and stirred for another 5 h. The resulting mixture was poured into a syringe and placed in the electrospinning apparatus with the NiTi wires stacked on the foil. The feeding rate of the syringe pump was set at 0.2 mL/h. The voltage of the electrospinning device was controlled at 20 kV. After 2 h, the nanofibers were wholly placed on the wires as a coating agent. After the electrospinning process, the samples were placed in the oven for 10 h, followed by furnace for 1 h at 400°C.

### 2.3. Antibacterial Procedure

**Figure 1:**
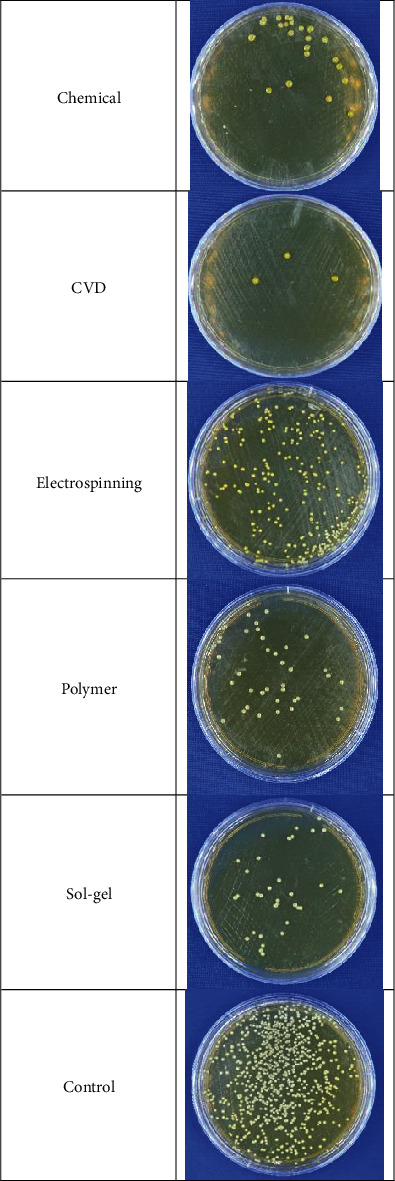
Colony count method.

Round 0.016-inch orthodontic NiTi straight wires (OrthoTechnology, FL, USA) with 18 cm length were cleaned with 70% ethanol solution three times and divided into nine 2 cm pieces. The antibacterial properties of the coated wires were assessed against *S. mutans* (ATCC 35668). Bacteria were cultured under the sterile condition on brain-heart infusion (BHI) agar at 37°C for 24 h. After the incubation period, the standard 0.5 McFarland concentration of bacteria containing approximately 1.5 × 10^8^ CFU/mL was prepared into the BHI broth. Under the sterile condition, 1 mL of the prepared concentration was inoculated to each of the microtubes containing solution synthesis, sol-gel, electrospinning, polymer samples, and control without ZnO NPs and incubated at 37°C for 24 h. After the incubation period, approximately 20 *μ*L from all tubes was transferred to an agar medium to study the microorganisms' growth. For each tube that showed the growth, several dilutions (10^−1^, 10^−2^, 10^−3^, etc.) were prepared to count the number of bacterial colonies. The spread plate technique was performed to count the number of colonies in the samples. A small volume of dilute microbial suspension (10 *μ*L) was inoculated into the center of an agar plate and spread evenly over the surface with a sterile bent-glass rod and incubated at 37°C for 24 h. After the incubation, the plate containing 30–300 cells was selected. Then, the number of colonies was counted and reported in CFU/mL (Figure 1).

To calculate the microbial cell reduction percentage (*R*, %), we used the following equation:

(1) $$\begin{array}{c} R\%=\frac{{\text{CFU}}_{\text{control}}-{\text{CFU}}_{\text{sample}}\;}{{\text{CFU}}_{\text{control}}}\times 100\%. \end{array}$$

CFU_control_ and CFU_sample_ are the numbers of colony-forming units per milliliter for the negative control sample (without ZnO) and the ZnO precipitation samples, respectively [25].

### 2.4. Characterization of NPs

To characterize the physical properties and surface morphologies of ZnO NPs, a field emission scanning electron microscope (FE SEM) (MIRA3 FEG-SEM Company: Tescan, Czech) equipped with energy-dispersive X-ray spectroscopy (EDX) was used. To identify the composition and track the formation of NPs, EDX analysis was used.

## 3. Results

### 3.1. Structure and Morphology

**Figure 2:**
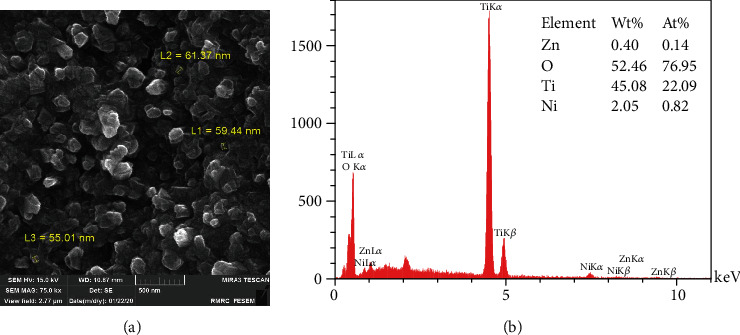
CVD method: (a) FE SEM images; (b) EDX analysis.

The FE SEM image in Figure 2(a) represents the morphology of ZnO NPs synthesized by the CVD technique, as it shows NPs within the range of 59-61 nm. The EDX analysis in Figure 2(b) revealed the composition of NPs as 0.14 At.% of Zn,76.95 At.% of O, 22.09 At.% of Ti, and 0.82 At.% of Ni.

**Figure 3:**
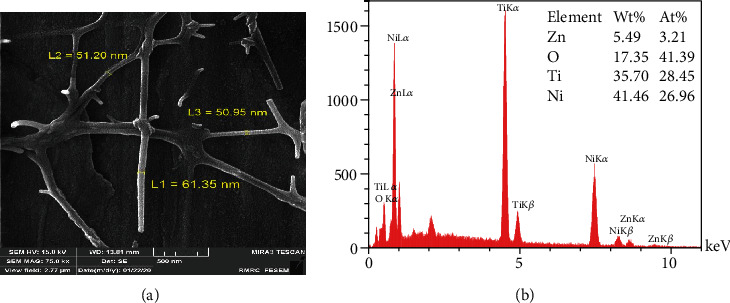
Electrospinning: (a) FE SEM images; (b) EDX analysis.

**Figure 4:**
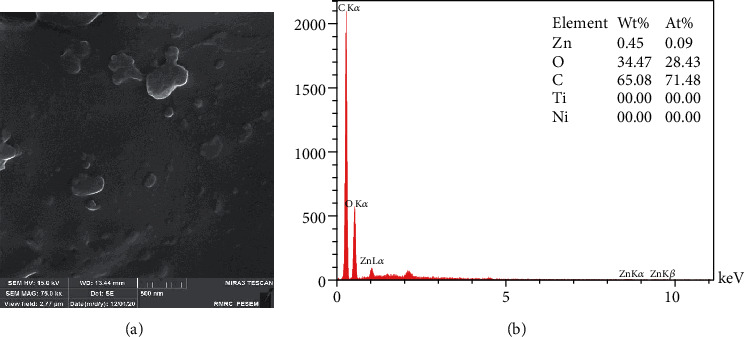
Polymer composite coating: (a) FE SEM images; (b) EDX analysis.

Figure 3(a), marked as electrospinning technique, presents an entirely different shape of particles. Electrospinning gave a branch of fibers gathered together in a network. The length of these fibers varies from 1 *μ*m to 1.5 *μ*m, and their diameter was in the range of 51-61 nm. The EDX spectrum (Figure 3(b)) confirmed the presence of Zn and O besides Ni and Ti with the At% of 3.21% and 41.39%, respectively.  Figure 4(a) confirms the presence of nonuniform and excursive ZnO NPs in nanocomposite matrices (PVA) that makes measuring the size of the particles impossible. EDX analysis shows that the At% of Zn was 0.09% and that of O was 28.43%., which was the lowest amount of Zn between groups and also without detection of Ti and Ni. This might indicate the excellent coverage of the NiTi wire with NPs. Presence of C is related to the alcohol of the PVA (Figure 4(b)).

**Figure 5:**
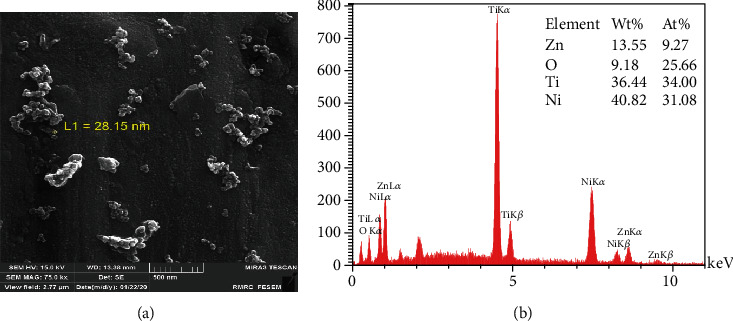
Sol-gel: (a) FE SEM images; (b) EDX analysis.

Figure 5(a) refers to the FE SEM image of ZnO NPs synthesized during the sol-gel synthesis process. Having the finest size of NPs than the previously mentioned samples with the approximate diameter of 28 nm, spherical particles tend to cluster together. Due to the EDX spectrum, the highest At% of Zn (9.27%) belongs to the sol-gel synthesis process (Figure 5(b)).

**Figure 6:**
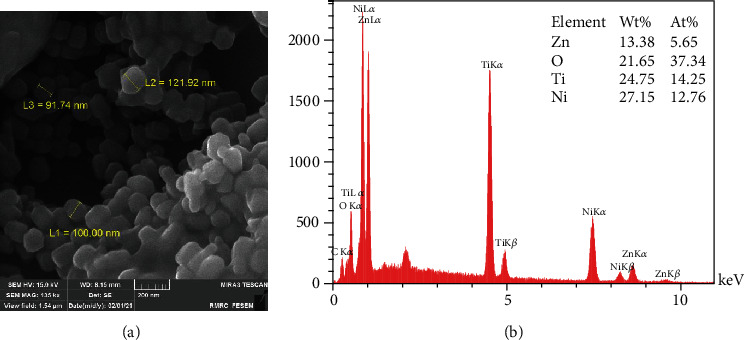
Chemical precipitation method: (a) FE SEM images; (b) EDX analysis.

As shown in the FE SEM image (Figure 6(a)), well-dispersed, uniform, and hexagonal NPs with spherical ends on either side of the particle are known as precipitation synthesis [20]. The scale of the particles is in the range of 30-150 nm, and the composition of the NPs (At%) as seen in EDX analysis is 5.65% for Zn and 37.34% for O (Figure 6(b)).

### 3.2. Antibacterial Activity

**Figure 7:**
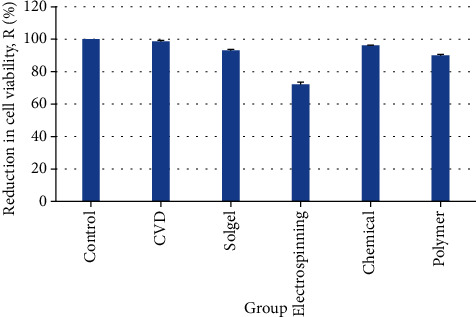
Reduction in cell viability for *S. mutans* bacteria after coating with ZnO particles in 5 groups.

A relatively high antibacterial effect was seen in all the samples containing ZnO NPs compared to the negative control group (without ZnO) by performing the colony count method.  Table 1 confirms the outcomes of the reduction in cell viability for the *S. mutans*. Considering the various dimensions and morphologies related to different ZnO particle synthesized methods, it is apparent that bacteriostatic activity results vary between the groups (Figure 7). The highest percentage of microbial cell reduction was 98.6%, which belonged to the vapor deposition method with high-density NPs in a subtle and well-dispersed pattern.

The precipitation method and sol-gel synthesis groups (with 96.14% and 93.05% microbial cell reduction, respectively) represented quite good antibacterial activity due to their small size and well-dispersed spherical NPs. The lowest percentage was 72%, which belonged to the electrospinning group.

## 4. Discussion

ZnO, as an antimicrobial agent, is investigated for both micro- and nanoscale. Nonetheless, the significant bactericidal effect is exhibited when the dimensions reduce in nanometer [38]. Three crystallized structures are typical for ZnO, known as wurtzite, zinc-blende, and rock salt. In a hexagonal wurtzite structure, four oxygen atoms surround each tetrahedral Zn atom and make it thermodynamically stable compared to the zinc-blende structure, which is metastable in an ambient environment and needs to be stabilized by growth techniques [39].

The nonspecific manner of ZnO NPs makes it challenging to interpret the primary mechanism of antibacterial function. The proposed mechanisms may include reactive oxygen species (ROS) formation, disruption of cell wall integrity, and release of Zn^2+^ ions. Whatever the function is, it is obvious that ZnO NPs act as bacteriostatic agents. Some of the differences among these particles are due to the shape, crystallinity, particle size, and amount of ZnO synthesized [40].

Morphology and particle size are two significant variables that affect the antibacterial behavior of ZnO NPs and should be precisely controlled [40]. By improving the surface characteristics of these particles, such as biocompatibility and bioconjugation, the NPs show great characteristics, such as modifying the surface properties and high stability of NPs [41].

In this study, the antibacterial activity of ZnO NPs was significantly improved when the surface area of particles increased, by the ZnO NPs prepared via the CVD coating method [34–37]. Castro-Mayorga et al. tested the antimicrobial effects of the hexagonal-pyramid (P-ZnO), star (S-ZnO), rod (R-ZnO), and porous ball (B-ZnO) particles synthesized via aqueous precipitation. Their results showed that the antibacterial properties of ZnO particles were in a direct relation with the specific surface area of particles and the hexagonal-pyramid NPs (P-ZnO) had the highest surface area and antibacterial effect [34].

Stanković et al. [25] synthesized various shapes and sizes of ZnO NPs via different kinds of stabilizing agents, including polyvinyl pyrrolidone (PVP), polyvinyl alcohol (PVA), and poly (*α*, *γ*,*ι*-glutamic acid) (PGA) in a low-temperature hydrothermal technique. They tested the antibacterial effects of ZnO NPs against *Escherichia coli* and *Staphylococcus aureus* through the colony count method and reported a reduction in cell viability, as in the current study. The PVA group represented the highest antibacterial activity against both microorganisms. A better reduction in cell viability of samples was shown, especially in PVA, which was similar to the current study except in the type of bacteria that was replaced with *S. mutans*, since our aim was to reduce WSLs and caries.

Talebian et al. [42] also examined the antibacterial behavior of ZnO NPs against *E. coli* and *S. aureus* via a simple solvothermal procedure without using any catalysts. In addition, they observed optical properties of the ZnO NPs by UV-vis absorption and photoluminescence. Flower-like, hexagonal rod-like, and spherical-like ZnO NPs were synthesized in water, 1-hexanol, and ethylene glycol, respectively. Due to the higher surface interstitial defects that reduce hole recombination, the flower-like NPs demonstrated excellent antibacterial behavior.

Another study conducted by Raghupathi et al. [43] evaluated the particle size-dependent antibacterial behavior of ZnO NPs. The results demonstrated significant antibacterial activity after decreasing particle size from 212 nm to 12 nm, and particles smaller than 12 nm had a bactericidal function. Deteriorating the bacterial cell wall was announced as the probable reason for this cell death.

Padmavathy and Vijayaraghavan [44] used disk diffusion assay to measure the antibacterial effect of ZnO NPs synthesized via two different methods. The increase of inhibition zone around ZnO NPs against *E. coli* may be due to the simplified ability of smaller NPs in rupturing the bacterial membrane. All samples' concentrations above 1 mM were bacteriostatic, and concentrations between 5 and 100 mM were bactericidal. The bactericidal behavior was better in 12 nm particles compared to 45 nm and 2 *μ*m particles.

Dutta et al. [45] studied the antibacterial activity of ZnO NPs and their correlation to different particle sizes and oxygen vacancies. Their results showed more bacterial inhibition (increased bacteriostatic activity) with smaller particle sizes since decreasing the particle size could enhance the surface-to-volume ratio. In addition, ZnO NPs with increased oxygen vacancies facilitated the interaction with a bacterial membrane containing a negative charge.

Similar to our study, Stanković et al. [25] and Talebian et al. [42] indicated the specific morphologies seen in FE SEM images close to their antibacterial activity. Larger specific surface area, small spherical and cuboid particles in sol-gel synthesis, precipitation method, and CVD can contact a greater number of *S. mutans* cells. The microbial cell reduction of 83.3% for the nanowires prepared by Wang et al. [46] with an approximate length of 1 *μ*m and a diameter of 150 nm is in good agreement with our nanofibers synthesized by electrospinning (with 1 *μ*m length and 61 nm and 72% reduction in microbial cell viability). The relatively high antibacterial activity of NPs synthesized by the sol-gel method is similar to the study directed by da Silva et al. [47] that showed high At% of Zn (9.27%) and O (25.66%) detected in the EDX spectrum. A close look at the EDX table reveals that low At% of Zn (0.09%) in particles stabilized by PVA may be the reason for lower antibacterial activity compared to other study groups. Increasing the At% of ZnO in polyvinyl alcohol matrices in further studies can probably enhance the bacteriocidic activity of ZnO NPs.

Although some previous studies attempted to show the antibacterial activity of ZnO NPs [25], few research has investigated the application of ZnO NPs in orthodontics to reduce the incidence of WSL [6, 48, 49]. In the current study, ZnO NPs exhibited bacteriostatic activity against *S. mutans*. Except for the study by Kachoei et al. in 2016 [17], none of the existing literature studied NiTi orthodontic wires. Similar to the current study, Kachoei et al. [17] investigated ZnO NPs synthesized by the chemical precipitation method; they used resazurin for antibacterial assay. The plates containing ZnO-coated wires had no color change, indicating no bacterial growth.

Chun et al. [49] evaluated the antibacterial effect of stainless steel (SS) wires coated with titanium oxide (TiO_2_) by the sol-gel method using the dilution agar plate method for *S. mutans* and spectrophotometry for *Porphyromonas gingivalis*. Coated wires showed bactericidal activity against *S. mutans* and *P. gingivalis*. Shah et al. [48] also studied the antibacterial properties of TiO_2_ NPs but coated on preadjusted SS brackets against *Lactobacillus acidophilus* instead of orthodontic wires. They used the radiofrequency (RF) magnetron sputtering method to coat the brackets. Their results indicated less bacterial mass around brackets and bactericidal effect of TiO_2_, which resulted in a lower rate of WSLs. Photocatalytic characteristics of TiO_2_ NPs make them more efficient in eliminating bacteria. However, unfortunately, these catalytic properties under UV light cause serious genetic harm in human cells and tissues, so the application of TiO_2_-coated wires and brackets in human beings is unsuitable.

Ramazanzade et al. [6] compared the antibacterial activity of CuO-, ZnO-, and CuO-ZnO-coated brackets against *S. mutans* with the colony count method. They also confirmed the bactericidal effect of NPs in all groups compared to the control group with no NPs. Also, CuO and ZnO-CuO groups had better antibacterial properties than ZnO-coated brackets on *S. mutans*. Even though Cu NPs have remarkable chemical, physical, and biological characteristics, their fast oxidation in front of air makes them an inappropriate choice in orthodontics.

In general, despite various studies demonstrating antimicrobial activity of NPs [40], the current study is the only one assessing the antibacterial properties of ZnO NPs in different 3D shapes and morphologies coated on NiTi wires. Interestingly, different morphologies resulted in different bacteriostatic properties based on the method used for synthesizing NPs. In addition to this attractive characteristic of ZnO NPs in reducing WSLs and caries during the first months of comprehensive orthodontic treatment [50], they also reduce the frictional coefficient of NiTi wires [17]. The high frictional forces while sliding the tooth for leveling and alignment of irregular teeth in the first stage of orthodontic treatment are significant disadvantages of these precious wires. NiTi wires have facilitated the clinical procedure and shortened the chair time of the first stage compared to multistrand and coaxial SS wires. Nevertheless, the overall duration of leveling and alignment stage is almost like multistrand and coaxial wires due to the frictional resistance to sliding [51]. Hence, ZnO NPs act as solid lubricants, smoothen the surface of NiTi wires, result in decreased frictional forces, and enhance the efficacy of these orthodontic wires [17].

## 5. Conclusion

In this study, chemical precipitation method, CVD, electrospinning, polymer composite coating, and the sol-gel method produced different forms and dimensions of ZnO nanostructures. FE SEM images demonstrated NPs with a diameter of 60 nm in CVD, fiber-like forms of electrospinning reaching 1.5 *μ*m in length, hexagonal particles within a wide range of particle scale in precipitation synthesis, amorphous nanostructures in polymer composite coating, and spherical NPs with about 28 nm in the sol-gel method. Regardless of different physicochemical properties of ZnO NPs, all samples showed excellent antimicrobial results against gram-positive coccus, *S. mutans*. The highest antibacterial effect with 98%, 96%, and 93% microbial cell reduction belonged to CVD, precipitation method, and sol-gel synthesis, respectively, and the lowest cell reduction was seen in the electrospinning method (72%). The apparent antibacterial activity of smaller NPs with the larger surface area makes these methods a precious way to produce NiTi archwires to protect teeth and reduce the rate of WSLs during orthodontic treatment.

## Figures and Tables

**Table 1:** The results of antibacterial properties of ZnO nanoparticles on the *S. mutans* bacterial culture (*R* (%) reduction in viability).

Group	Control	Number × 10^8^	*R* (%)
CVD	324 × 10^8^	4.5 (2.12)	98.61 (0.65)
Sol-gel		22.5 (2.12)	93.05 (0.65)
Electrospinning		90.5 (4.95)	72.07 (1.53)
Chemical		12.5 (0.71)	96.14 (0.22)
Polymer		32.5 (2.12)	89.97 (0.65)

The results stand for mean (SD: standard deviation, *n* = 2).

## Data Availability

Data supporting the conclusion of this article are within the manuscript.
